# Secondary Deinococcus proteolyticus Infection Following Varicella-Zoster Virus Infection in a Child: A Case Report Describing a Rare Medical Condition

**DOI:** 10.7759/cureus.100586

**Published:** 2026-01-01

**Authors:** Mariam M Elmakkawy, Sithra Rengasamy, Kang Nien Ho, Nur Liyana Abd Wahab

**Affiliations:** 1 Department of Pediatrics, Hospital Sultan Abdul Aziz Shah, University Putra Malaysia, Selangor, MYS; 2 Department of Dermatology, Hospital Sultan Abdul Aziz Shah, University Putra Malaysia, Selangor, MYS

**Keywords:** deinococcus proteolyticus, necrotizing skin lesion, opportunistic pathogen, pediatric infection, post-varicella

## Abstract

*Deinococcus proteolyticus* belongs to Deinococcaceae, a family of bacteria characterized by an exceptional ability to withstand the lethal effects of DNA-damaging agents, including ionizing radiation, ultraviolet (UV) light, and desiccation. It was isolated from the Sahara surface sands, an extreme and nutrient-poor environment, regularly exposed to intense UV radiation, cycles of extreme temperatures, and desiccation. *D. proteolyticu*s infection has seldom been implicated in human infections. The child presented with multiple necrotic skin lesions following a varicella infection, later complicated by *D. proteolyticus* bacteremia. The patient responded well to targeted antibiotic therapy and surgical debridement, achieving complete recovery. The literature on *D. proteolyticus* as a human pathogen is limited, and this case underscores its potential clinical significance. Although its occurrence is extremely rare, clinicians should recognize it as a possible opportunistic pathogen in severe cutaneous infections to ensure timely and appropriate management.

## Introduction

Secondary bacterial infection is a well-recognized complication of varicella-zoster virus (VZV) infection, arising from disruption of the epidermal barrier and virus-induced cytopathic injury. The virus causes degeneration of keratinocytes and loss of epidermal integrity, creating areas of superficial erosion that are highly susceptible to colonization and invasion by bacterial pathogens. In addition, VZV induces a transient immunosuppressive state characterized by lymphopenia, reduced natural killer (NK) cell activity, and temporary bone marrow suppression, all of which impair the host’s ability to contain secondary microbial invasion. These combined effects, breakdown of the physical skin barrier together with transient immune dysregulation, provide an optimal environment for opportunistic organisms to proliferate, transforming otherwise self-limiting skin lesions into sites of bacterial superinfection. Children are particularly vulnerable to this complication, as their immune responses to viral infections are still developing, and their skin lesions are often more widespread and prone to excoriation [1].

*D. proteolyticus* is a Gram-positive, non-spore-forming, pigmented bacterium belonging to the genus Deinococcus, renowned for its extreme resistance to radiation, desiccation, and oxidative stress. While members of this genus have been studied extensively for their resilience mechanisms and biotechnological applications [2].

Deinococcus species are unique among Gram-positive bacteria in that their cell wall architecture includes both a thick peptidoglycan layer and an outer membrane analogous to that of Gram-negative organisms. This dual-envelope structure, together with intracellular carotenoid pigments and efficient DNA repair systems, accounts for their remarkable resistance to radiation and desiccation. Understanding this atypical envelope composition helps interpret both their staining variability and their broad antibiotic susceptibility profile observed in this case. Although widely distributed in the environment, including soil, air, and hospital surfaces, these organisms are rarely pathogenic to humans. Reports of human infections involving Deinococcus species are exceedingly uncommon, and Deinococcus proteolyticus has only rarely been identified in clinical isolates, with its pathogenic potential remaining largely unknown [3].

The genus Deinococcus was first described in 1956 following the discovery of *Deinococcus radiodurans*, one of the most radiation-resistant organisms known. The genus includes over 50 species, most of which inhabit extreme environments such as deserts, radioactive sites, and arid soils. *D. proteolyticus* was isolated from the feces of Lama glama and characterized as an orange-red pigmented, nonmotile coccus with remarkable stress tolerance. Despite its robust physiology, *D. proteolyticu*s remains primarily an environmental organism with no established role in human pathology [2,3].

*D. proteolyticus* belongs to the phylum Deinococcota, class Deinococci, and order Deinococcales. The species shares extensive phylogenetic similarity with *D. radiodurans* and *D. geothermali*s, reflecting evolutionary conservation of stress-response genes. Comparative analysis of the DnaK and GroEL protein sequences further confirms their close taxonomic relationship within the genus [4].

*D. proteolyticus* appears as spherical or slightly ovoid cells, typically forming tetrads. Colonies are orange-red due to carotenoid pigments that provide photoprotection and antioxidative defense. It is an aerobic, catalase-positive, oxidase-positive organism capable of surviving harsh conditions, including oxidative and radiation stress. Optimal growth occurs at 30-37°C on nutrient-rich media. The species name “proteolyticus” reflects its early characterization as a protease-producing bacterium [5].

The complete genome of *D. proteolyticus* MRPᵀ was sequenced and described by Copeland et al. The genome comprises approximately 2.7 Mbp with a GC content of 66%. It exhibits high redundancy in genes involved in DNA repair (RecA, PolA, PprA), antioxidant defense (superoxide dismutase, catalase), and stress response (GroEL, DnaK). Carotenoid biosynthetic genes are responsible for the organism’s pigmentation and radiation resistance. These genomic traits underlie the organism’s exceptional resilience to ionizing radiation and desiccation [2].

*D. proteolyticus* has been isolated from soil, animal feces, and industrial environments, reflecting its adaptability to diverse ecological niches. The organism’s oxidative stress resistance has inspired its use in recombinant studies aimed at improving bacterial tolerance. For instance, Yang et al. demonstrated that a *D. proteolyticus* genomic library conferred enhanced oxidative stress resistance and polyhydroxybutyrate production in *Escherichia coli*. Additionally, its robust DNA repair mechanisms make it a model organism in studies of radiation protection and stress physiology [5].

Identification of *D. proteolyticus* typically requires molecular methods, including 16S ribosomal RNA gene sequencing or matrix-assisted laser desorption ionization-time of flight mass spectrometry (MALDI-TOF MS). However, clinical identification may be challenging due to limited reference data in microbial databases. When isolated from patient specimens, true infection should only be considered if recovered from a sterile site, supported by clinical correlation, and reproducible in culture [6].

We present a rare and previously undescribed pediatric case of secondary *D. proteolyticus* infection occurring in the context of VZV infection. To our knowledge, this is the first documented report implicating *D. proteolyticus* as a pathogenic organism in a human host.

## Case presentation

**Figure 1:**
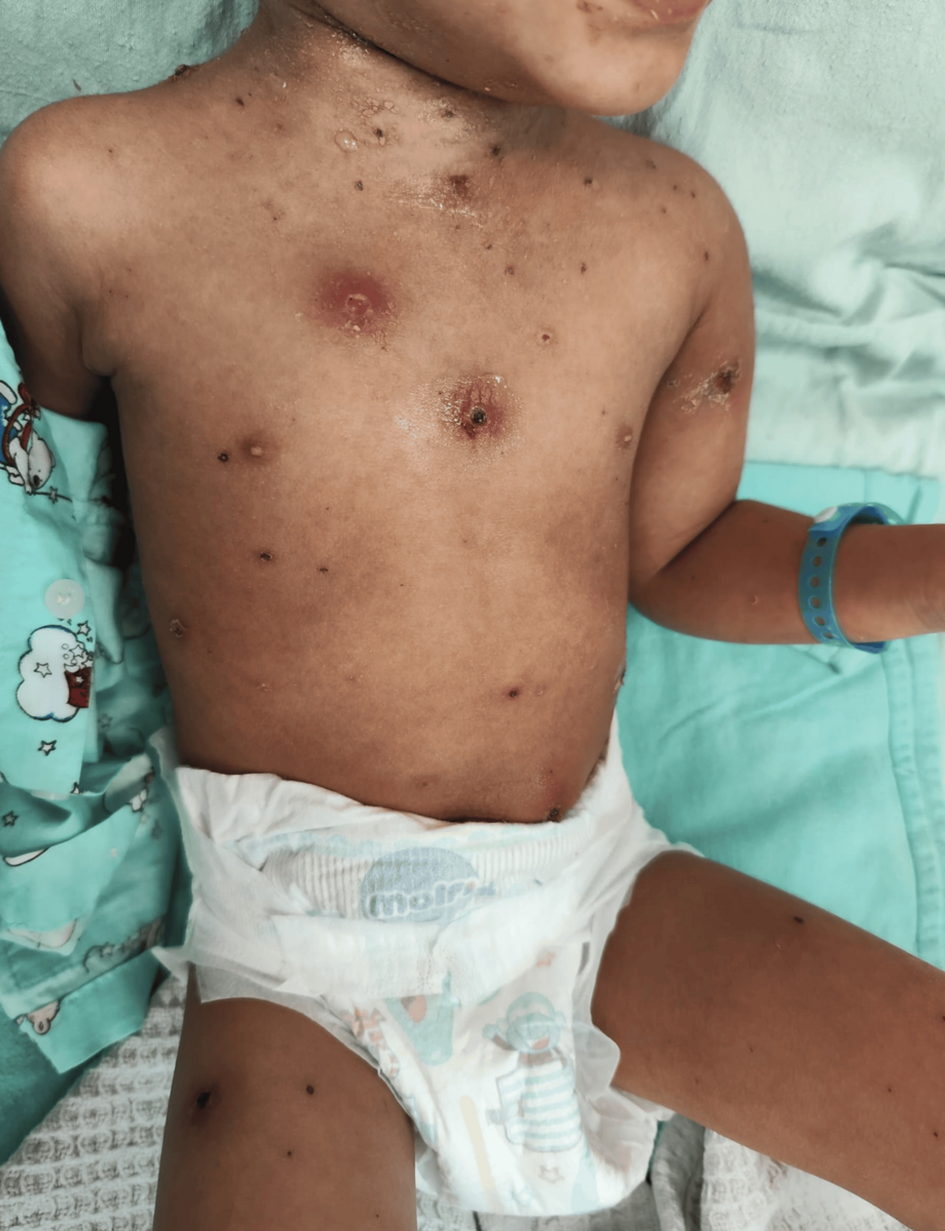
Multiple crusted, erosive, and necrotic lesions observed over the left shoulder, anterior chest wall, neck, and abdominal wall. The appearance was most consistent with post-varicella ulceration with secondary bacterial infection

**Figure 2:**
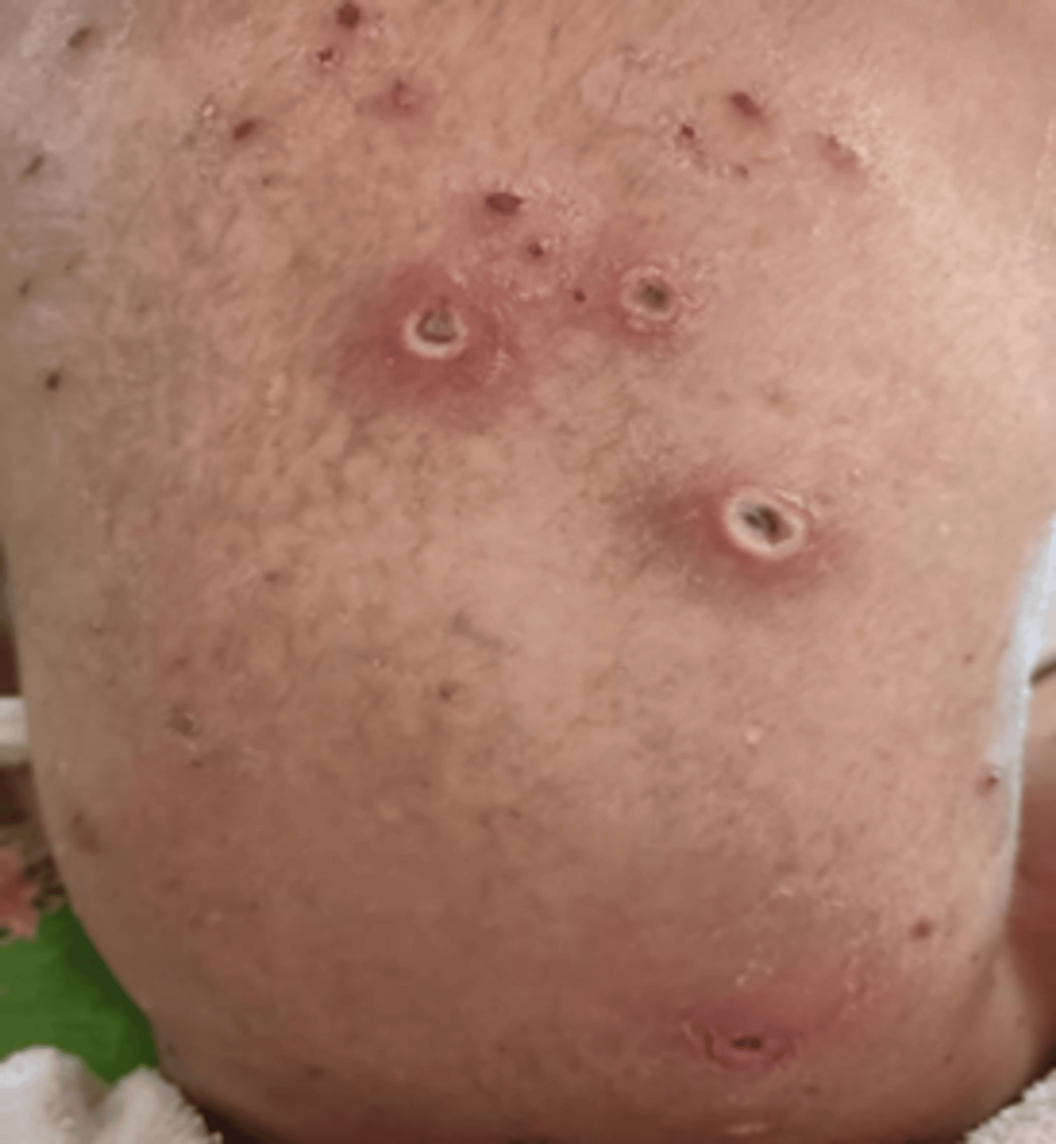
Multiple crusted and necrotic ulcerative lesions were present across the back, compatible with post-varicella ulceration with secondary bacterial involvement

A three-year-old Malay boy presented with a six-day history of persistent fever and progressive skin lesions following exposure to siblings with chickenpox. He had been clinically diagnosed with varicella-zoster early in the illness. By the third day of infection, the fever worsened, and he developed widespread vesicular eruptions that evolved into ulcerated necrotic lesions with surrounding erythema. There was no prior medical illness, immunodeficiency, or hospital admission history. On physical examination, the child was febrile, irritable, and underweight for age. Multiple crusted and necrotic lesions were observed over the left shoulder, proximal arm, anterior chest wall, neck, right shoulder, and abdominal wall (Figures 1, 2).

**Figure 3:**
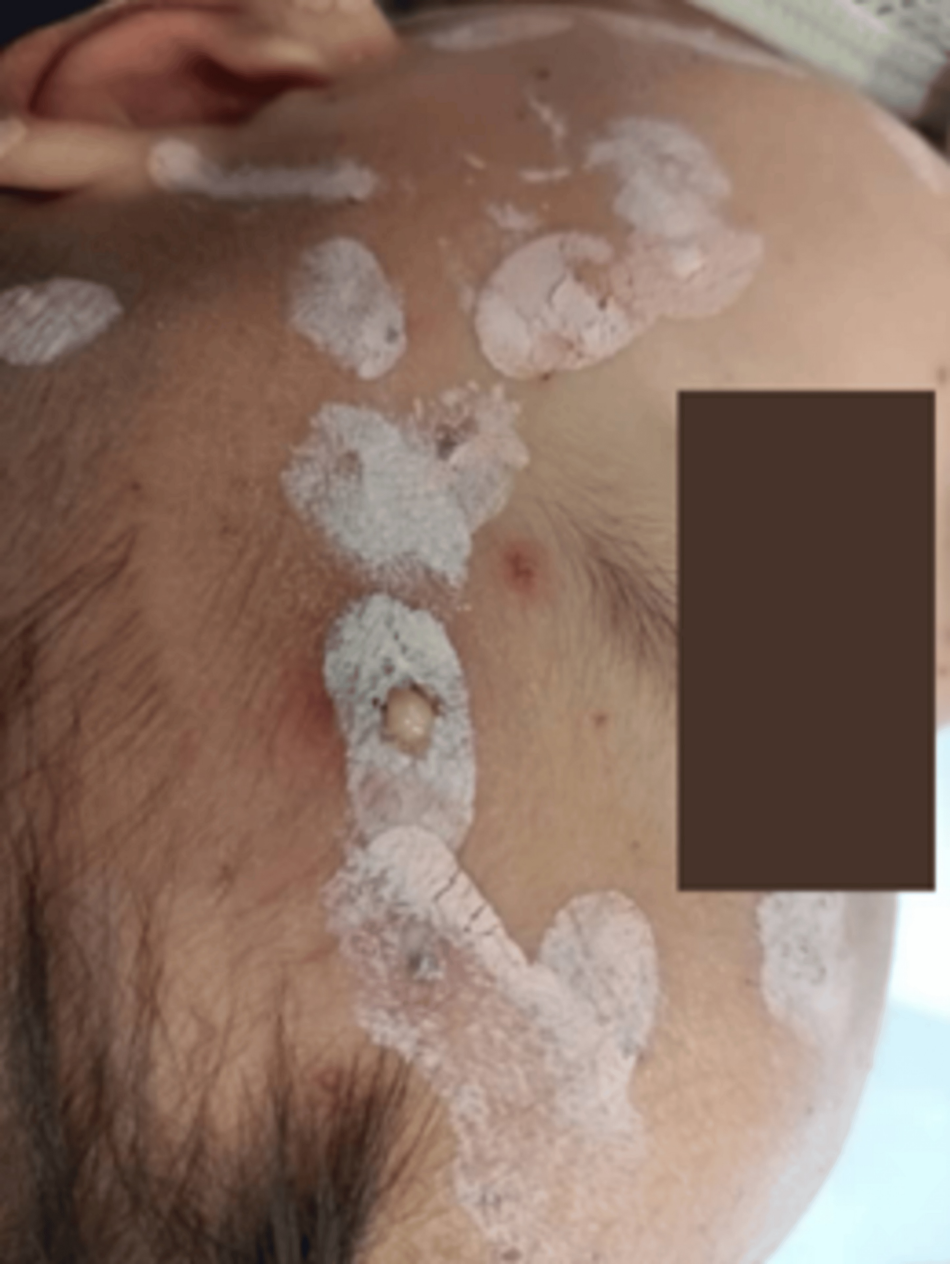
One of the lesions over the forehead appeared fluctuant and was oozing purulent material, suggesting localized secondary bacterial infection

Some lesions were fluctuant and oozing pus (Figure 3).

Initial laboratory investigations revealed leukocytosis (white blood cell count of 26.69 × 10³/µL), with a differential count showing neutrophilia (75.1%) and mild lymphopenia (17.4%; absolute lymphocyte count 4.65 × 10³/µL). A nasopharyngeal swab for severe acute respiratory syndrome coronavirus 2 real-time reverse-transcriptase polymerase chain reaction was performed. It yielded a negative result, excluding coronavirus disease 2019 infection as a cause of lymphopenia.

The substantially elevated inflammatory markers observed in this case offer critical diagnostic insight. C-reactive protein (CRP) levels exceeding 100 mg/L, as demonstrated in this patient with a value of 191 mg/L, are strongly indicative of bacterial superinfection, rather than an isolated viral process. Likewise, the markedly increased erythrocyte sedimentation rate (ESR) of 67 mm/hour is consistent with a sustained systemic inflammatory response, further supporting the presence of secondary bacterial involvement.

His hemoglobin concentration was 10.6 g/dL, and his hematocrit was 32.2%, both falling below the established pediatric reference ranges. The anemia is most plausibly multifactorial, attributable to the acute VZV infection, which is known to induce transient bone marrow suppression in children. Additionally, a robust systemic inflammatory response may have contributed to anemia of acute illness, mediated by impaired erythropoiesis and altered iron metabolism. A reduction in oral intake during the acute phase of illness may have served as an additional contributing factor. Collectively, these mechanisms likely explain the transient decline in hemoglobin levels observed at presentation.

Immunological screening, including human immunodeficiency virus, viral hepatitis serology, and immunoglobulin levels, was unremarkable. Lymphocyte subset enumeration demonstrated a mildly decreased proportion of NK cells (6.78%, ref. 8%-15%), suggesting a transient or mild immunocompromised state. Renal and hepatic profiles were unremarkable apart from mild hypoalbuminemia (albumin level was 23 g/L), while total protein was preserved. These findings collectively indicated a systemic inflammatory process with mild immune suppression (Table 1).

**Table 1:** Summary of laboratory test results on the first week of admission NK: natural killer; IgG: immunoglobulin G

Category/test (unit)	Observed value	Normal range
Hematology (full blood count + differential)		
White blood cell count (×10³/µL)	26.69	5.0-15.0
Red blood cell count (×10⁶/µL)	4.37	4.00-5.20
Hemoglobin (g/dL)	10.6	11.0-14.0
Hematocrit (%)	32.2	34.0-40.0
Mean corpuscular volume (fL)	73.7	75.0-87.0
Mean corpuscular hemoglobin (pg)	24.3	24.0-30.0
Mean corpuscular hemoglobin concentration (g/dL)	32.9	31.0-37.0
Platelet count (×10³/µL)	591	200-490
Red cell distribution width (%)	14.0	11.6-14.0
Neutrophils (%)	75.1	40.0-75.0
Lymphocytes (%)	17.4	20.0-40.0
Monocytes (%)	4.3	2.0-10.0
Eosinophils (%)	3.1	1.0-6.0
Basophils (%)	0.2	0-2
Neutrophil count (×10³/µL)	20.04	1.50-8.00
Lymphocyte count (×10³/µL)	4.56	6.00-9.00
Monocyte count (×10³/µL)	1.11	0.20-1.00
Eosinophil count (×10³/µL)	0.83	0.10-1.00
Basophil count (×10³/µL)	0.06	0.02-0.10
Liver function tests		
Total protein (g/L)	67	56-75
Albumin (g/L)	23	38-54
Total bilirubin (µmol/L)	6.1	<22.0
Alanine transaminase (U/L)	8	10-50
Aspartate transaminase (U/L)	19	10-50
Alkaline phosphatase (U/L)	116	142-335
Inflammatory markers		
C-reactive protein (mg/L)	191	<5.0
Erythrocyte sedimentation rate (mm/hour)	67	<10
Lymphocyte subset enumeration (flow cytometry)		
Total T cells (CD3) (%)	71.87	62-69
Total T cells (CD3) (×10⁶/L)	3,353	1,800-3,000
Total B cells (%)	20.51	21-28
Total B cells (×10⁶/L)	957	700-1,300
Helper T cells (CD4) (%)	36.95	30-40
Helper T cells (CD4) (×10⁶/L)	1,723	1,000-1,800
Cytotoxic T cells (CD8) (%)	27.33	25-32
Cytotoxic T cells (CD8) (×10⁶/L)	1,275	800-1,500
NK cells (%)	6.78	8-15
NK cells (×10⁶/L)	316	200-600
CD4/CD8 ratio	1.34	1.0-2.0
IgG subclasses (g/L)		
IgG1	4.384	2.24-8.03
IgG2	0.960	0.52-2.31
IgG3	0.708	0.16-1.08
IgG4	0.608	0.02-0.87

Empirical intravenous cloxacillin was initiated to treat a presumed secondary bacterial skin infection. As the child remained febrile after 48 hours, therapy was escalated to cefazolin combined with amikacin to broaden coverage against both Gram-positive and Gram-negative organisms.

The dermatology team subsequently reviewed the lesions and considered them most consistent with post-varicella ulcerative changes. The ulcerations were interpreted as part of the cytopathic healing phase of varicella-zoster infection, with secondary bacterial involvement contributing to delayed resolution. In view of the potential for environmental bacterial contamination, given the history of possible soil exposure through the family’s babysitter, who worked as a gardener, piperacillin-tazobactam was added to ensure adequate extended-spectrum coverage while awaiting final culture results.

Blood samples were collected aseptically from a peripheral vein before antibiotic administration and inoculated into BACTEC aerobic and anaerobic blood culture bottles (Becton Dickinson, USA). The bottles were incubated at 37°C for up to five days. When a bottle signaled positive at 48 hours, the contents were subcultured onto 5% sheep blood agar, MacConkey agar, and chocolate agar (Oxoid™, UK) and incubated aerobically at 37°C.

After 48 hours, small, smooth, orange-red colonies were observed on blood agar, with no growth on MacConkey agar and light growth on chocolate agar. Gram staining revealed Gram-positive cocci arranged mainly in tetrads. The isolate was catalase-positive, oxidase-positive, nonmotile, and non-spore-forming.

Definitive identification was achieved using MALDI-TOF MS (Premier Integrated Labs, Malaysia), which identified the organism as *D. proteolyticus *with a confidence score exceeding 99%.

The diagnosis of secondary *D. proteolyticu*s infection complicating VZV infection was concluded based on the organism’s isolation from blood culture, supported by the patient’s clinical presentation, disease progression, and subsequent improvement with targeted antimicrobial therapy. Antimicrobial susceptibility testing was performed via an automated microdilution system following Clinical and Laboratory Standards Institute guidelines, and the isolate was found to be susceptible to all antibiotics tested, including ampicillin, ceftriaxone, gentamicin, imipenem, linezolid, and vancomycin. A summary of susceptibility interpretations is shown in Table 2.

**Table 2:** Antimicrobial susceptibility testing S: susceptible The culture report was validated by two clinical laboratory scientists and a consultant medical microbiologist, confirming the authenticity of the findings (Premier Integrated Labs, Malaysia; Report ID: BGL25003038, January 2025)

Antibiotic	Result
Amoxicillin/clavulanic acid	S
Ampicillin	S
Ceftriaxone	S
Cefuroxime	S
Gentamicin	S
Imipenem	S
Linezolid	S
Piperacillin/tazobactam	S
Vancomycin	S
Rifampicin	S
Cotrimoxazole	S
Tetracycline	S

**Figure 4:**
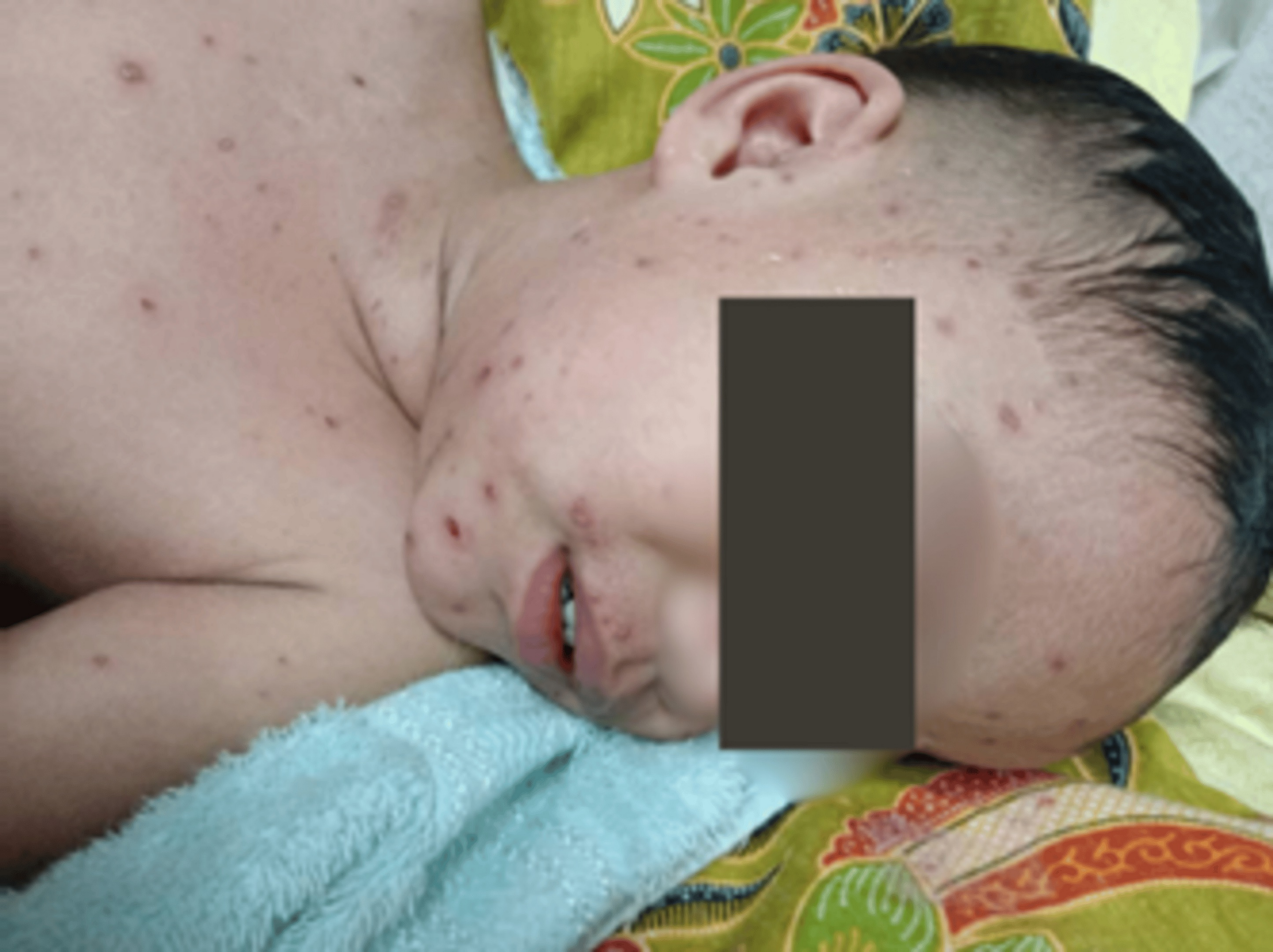
Swelling over the anterior neck with restricted neck movement

On the fifth day of admission, the child developed multiple new swellings that appeared over the anterior neck with restricted neck movement and tenderness (Figure 4).

Ultrasound imaging of the neck and upper limbs demonstrated diffuse subcutaneous thickening and edema consistent with cellulitis, but no abscess formation.

Despite ongoing antimicrobial therapy, the skin lesions over the left arm and chest subsequently worsened, progressing to purulent discharge and areas of necrosis. Surgical intervention was deemed necessary, and the patient underwent debridement with removal of necrotic tissue from the left shoulder and proximal arm, followed by incision and drainage of an abscess identified intraoperatively. Further imaging of the abdomen and chest revealed no intra-abdominal abscesses, deep collections, or evidence of osteomyelitis. Throughout this period, the child remained hemodynamically stable. Although he continued to experience a persistent low-grade fever, his clinical condition improved following surgical source control and optimization of antimicrobial therapy.

The patient completed eight days of intravenous cefazolin and seven days of piperacillin-tazobactam, followed by an oral cephalosporin course. His inflammatory markers markedly improved (CRP decreased from 191 to 9.9 mg/L, and ESR improved from 67 to 13 mm/hour), and leukocyte counts returned to normal.

He underwent wound care, and the skin lesions progressively healed without residual necrosis or scarring. The patient was discharged afebrile, with well-healed wounds, and follow-up confirmed complete recovery without recurrence.

## Discussion

Secondary bacterial infection is a well-recognized complication of VZV infection, arising from disruption of the epidermal barrier and virus-induced cytopathic injury. In this case, the child presented with multiple ulcerative and crusted lesions that were fully consistent with post-varicella ulceration and delayed healing, rather than a primary necrotizing bacterial dermatosis. The morphology and distribution of the lesions corresponded to the expected evolution of VZV lesions, from vesicles to erosions and ulceration, followed by crusting and secondary bacterial involvement. The progression of the lesions, together with the absence of features such as deep tissue necrosis, eschar formation, or vasculitic changes, supports the interpretation that the primary pathology was viral, with bacterial invasion occurring only after the integrity of the skin barrier had been compromised [1].

Although *D. proteolyticus* is not commonly associated with human disease, isolation of this environmental organism from a sterile blood culture specimen is clinically significant. Species within the Deinococcus genus possess remarkable resistance to oxidative stress, desiccation, and environmental extremes, allowing them to persist on surfaces such as soil, dust, and dehydrated skin. In this patient, exposure to soil and plant material through a household gardener provided a plausible environmental source of inoculation. Once the skin barrier was damaged by varicella-related cytopathic effects, the organism may have gained access to deeper tissues and the bloodstream. Its survival on dry, crusted lesions and its ability to withstand oxidative immune mechanisms likely contributed to its pathogenic potential in this setting [2-6].

The child’s transient immunological vulnerability further increased susceptibility to opportunistic infection. Laboratory studies demonstrated anemia, mild lymphopenia, and a reduction in NK cell populations, findings consistent with temporary bone marrow suppression and immune dysregulation commonly observed in acute VZV infection. Elevated inflammatory markers, particularly the markedly raised CRP (191 mg/L) and ESR (67 mm/hour), strongly supported the presence of bacterial superinfection rather than isolated viral disease. These inflammatory abnormalities, combined with clinical deterioration on day 5 of illness, indicated a transition from uncomplicated varicella skin lesions to a more extensive soft-tissue bacterial infection.

The microbiological evaluation further strengthened the diagnosis. The blood culture signaled positive at 48 hours, and subculture yielded Gram-positive cocci arranged in tetrads. MALDI-TOF MS subsequently identified the isolate as *D. proteolyticus* with a confidence score exceeding 99%. Although repeat cultures and molecular sequencing would have enhanced diagnostic certainty, the isolation of this organism from blood, a normally sterile site, along with the patient’s clinical response to targeted antimicrobial therapy, supports its interpretation as a true pathogen rather than a contaminant. Importantly, worsening lesions, the development of new swellings, and progression to purulent soft-tissue infection before therapy escalation are consistent with active bacterial disease. The improvement following surgical debridement and the addition of broad-spectrum antibiotics further validates the clinical relevance of this organism in the patient’s disease course.

This case underscores several key clinical considerations. First, secondary bacterial infection in the setting of varicella can involve atypical or rare pathogens, particularly when environmental exposure coincides with transient immune dysregulation. Second, skin lesions following varicella may exhibit significant ulceration and delayed healing, and their appearance can mimic other dermatological conditions if not interpreted in the context of recent viral illness. Third, identification of uncommon environmental organisms in sterile-site cultures should prompt balanced clinical judgment; while laboratory contamination must be considered, premature dismissal can delay necessary treatment. In this instance, a comprehensive microbiological assessment, including culture on multiple media, Gram staining, biochemical profiling, and MALDI-TOF MS, enabled accurate identification of the organism.

Ultimately, this represents one of the few reported pediatric cases of *D. proteolyticus* infection in the literature. It highlights the capacity of environmental bacteria to cause clinically significant infection in children with temporary immunological vulnerability following viral illness. The case reinforces the importance of correlating microbiological findings with clinical progression, radiological evaluation, and treatment response to arrive at a meaningful diagnosis. Moreover, the child’s complete recovery following combined surgical and antimicrobial management demonstrates that early recognition and multidisciplinary care are essential for favorable outcomes in rare opportunistic infections associated with varicella.

## Conclusions

This case highlights an uncommon instance of secondary *D. proteolyticus* infection complicating varicella-zoster virus infection in a child, expanding the limited knowledge of this environmental organism as a potential opportunistic human pathogen. The combination of virus-induced epidermal disruption, transient immune dysregulation, and possible environmental exposure likely facilitated bacterial invasion and subsequent bacteremia. Accurate identification of *D. proteolyticus* through culture and MALDI-TOF MS, together with careful clinical correlation, was essential to establishing its pathogenic role. The patient’s favorable outcome following timely surgical debridement and targeted antimicrobial therapy underscores the importance of maintaining a high index of suspicion for atypical bacterial pathogens in children presenting with complicated varicella lesions. This case reinforces the need for comprehensive microbiological evaluation and interdisciplinary management in pediatric soft-tissue infections, particularly when clinical progression is unusual or when environmental organisms are isolated from sterile sites. Early recognition, appropriate antibiotic coverage, and timely surgical intervention are crucial for favorable outcomes.
